# Errors in the 2017 APA Clinical Practice Guideline for the Treatment of PTSD: What the Data Actually Says

**DOI:** 10.3389/fpsyg.2017.01425

**Published:** 2017-08-22

**Authors:** Sarah K. Dominguez, Christopher W. Lee

**Affiliations:** ^1^School of Psychology and Exercise Science, Murdoch University, Perth WA, Australia; ^2^Faculty of Health and Medical Sciences, The University of Western Australia, Perth WA, Australia

**Keywords:** PTSD, EMDR, American Psychological Association, treatment guidelines, data analysis

## Abstract

The American Psychological Association (APA) Practice Guidelines for the Treatment of Posttraumatic Stress Disorder (PTSD) concluded that there was strong evidence for cognitive behavioral therapy (CBT), cognitive processing therapy (CPT), cognitive therapy (CT), and exposure therapy yet weak evidence for eye movement desensitization and reprocessing (EMDR). This is despite the findings from an associated systematic review which concluded that EMDR leads to loss of PTSD diagnosis and symptom reduction. Depression symptoms were also found to improve more with EMDR than control conditions. In that review, EMDR was marked down on strength of evidence (SOE) for symptom reduction for PTSD. However, there were several problems with the conclusions of that review. Firstly, in assessing the evidence in one of the studies, the reviewers chose an incorrect measure that skewed the data. We recalculated a meta-analysis with a more appropriate measure and found the SOE improved. The resulting effect size for EMDR on PTSD symptom reduction compared to a control condition was large for studies that meet the APA inclusion criteria (SMD = 1.28) and the heterogeneity was low (*I*^2^= 43%). Secondly, even if the original measure was chosen, we highlight inconsistencies with the way SOE was assessed for EMDR, CT, and CPT. Thirdly, we highlight two papers that were omitted from the analysis. One of these was omitted without any apparent reason. It found EMDR superior to a placebo control. The other study was published in 2015 and should have been part of APA guidelines since they were published in 2017. The inclusion of either study would have resulted in an improvement in SOE. Including both studies results in standard mean difference and confidence intervals that were better for EMDR than for CPT or CT. Therefore, the SOE should have been rated as moderate and EMDR assessed as at least equivalent to these CBT approaches in the APA guidelines. This would bring the APA guidelines in line with other recent practice guidelines from other countries. Less critical but also important, were several inaccuracies in assessing the risk of bias and the failure to consider studies supporting strong gains of EMDR at follow-up.

## Introduction

The American Psychological Association (APA) is acknowledged globally as an evidence based organization to support clinical practice. The organization aims to “*advance the creation, communication and application of psychological knowledge to benefit society and improve people’s lives*” (American Psychological Association, 2017b) and has as one of its five core values “*Knowledge and its application based upon methods of science”* (American Psychological Association, 2017a). APA treatment guidelines are regularly referred to in the literature with some documents published by the organization having hundreds or even thousands of citations (American Psychological Association, 1995; Wilkinson, 1999; American Psychological Association Zero Tolerance Task Force, 2008). Therefore, it is crucial that the organization ensures that it maintains the highest standards in scientific methodology, and is unbiased and apolitical in it’s reporting of guidelines for clinical practice. The latest guidelines do not meet those standards (Courtois et al., 2017, Unpublished).

The APA Practice Guideline Development Panel for the Treatment of Posttraumatic Stress Disorder (PTSD) was formed to review current data regarding the treatment of PTSD. The panel made recommendations based on a systematic review of the evidence for treatment for PTSD conducted by the Research Triangle Institute – University of North Carolina Evidence-Based Practice Center (RTI-UNC) (Jonas et al., 2013). The review found that EMDR was effective in decreasing PTSD symptoms, and achieving loss of diagnosis. EMDR was also effective in treating comorbid depression within the PTSD population. Despite this empirical support for EMDR, APA guidelines concluded that the strength of evidence (SOE) for EMDR to was low, while the SOE for other treatment approaches was classified as moderate to high. This paper identifies key methodological errors in the RTI-UNC paper with regards to the analysis of EMDR. Following this, additional analyses were conducted, correcting for these errors to give a more accurate view of the current empirical support for EMDR in treating PTSD.

## An Inappropriate Measure was Used to Determine Effect Size in an Included Study (Carlson et al., 1998)

The RTI-UNC review (Figure 17) referred to mean changes in PTSD symptoms for EMDR versus control comparisons. There are four studies listed and changes were assessed in each of the studies on identified primary measures. For example, in the Rothbaum et al. (2005) study, this was the Clinician Administered PTSD Scale (CAPS). The primary outcome measure for the Carlson et al. (1998) study was also the CAPS and this is reported in the original article for pre- and follow-up data. The effect size is large (Cohen’s *d* = 1.8). However, CAPS scores were not collected at post-treatment. A battery of self-report measures were collected at post-treatment including the Mississippi Scale for Combat Related PTSD (M-PTSD) and the Impact of Events Scale (IES). In the RTI-UNC analysis, the IES was chosen above the M-PTSD. Why is difficult to fathom. The M-PTSD is more comprehensive than the IES and was designed specifically to assess PTSD in veteran populations, which is the population involved in the Carlson study, and similar to the CAPS it is based on the DSM. Also, two memories were treated in this study, and the status of the memory focused on in the IES is unknown. That is, one memory was treated until 0–2 SUD was reached, and then treatment began on the next memory, but not necessarily finished, during the 12 sessions. Hence, the more global measures -CAPS and M-PTSD- are more appropriate. Finally, a review article at the time recommended the M-PTSD above all other self-report measures for assessing PTSD (Watson, 1990).

Initially, when comparing relaxation to EMDR the RTI-UNC reviewers report that they conducted meta-analyses using both measures (see Table 7). However, when they were describing which studies were included in their analysis, and wanted to compare the severity of PTSD symptoms at baseline for each study, they chose the M-PTSD over the IES (see Tables 9, 18). Also later in the report when assessing the effectiveness of relaxation, they again use the M-PTSD (p. 70). Why they reverted to the IES in the middle of the report when assessing change in the PTSD symptom level for this study is perplexing.

Changing the outcome measure from the IES to the M-PTSD significantly effects the results with regards to PTSD symptom reduction following EMDR. We entered this corrected data into Comprehensive Meta-Analysis Software and showed if this adjustment was made the effect size, precision, and consistency are all improved [SMD, -1.28 (-1.81 to -0.74); *I*^2^= 43%].

RTI-UNC guidelines define precision as the width of the confidence interval. Consistency is defined as the number of studies in the same direction and appears to take into account the heterogeneity (The RTI-UNC quote heterogeneity when discussing consistency in Appendix 1). Therefore heterogeneity at 43% for EMDR is better than mixed cognitive behavioral therapy (CBT), cognitive therapy (CT), and cognitive processing therapy (CPT) where heterogeneity was significant and ranged between 80 and 87%. In addition to EMDR being more consistent the precision improves to 1.07 (difference between lower and upper end of the confidence interval), which is better than both CPT (1.1) and CT (1.38). Therefore, there is no basis to argue SOE is better for these CBT therapies.

Changing the outcome measure analyzed to the more comprehensive measure of the M-PTSD provides a result more consistent with the rest of the data from the study. The effect size for the IES is small (SMD = -0.18) while the M-PTSD effect size is large (*d* = 1.01). The effect size for the CAPS at follow-up was large (*d* = 1.82) for the EMDR treatment compared to control condition, and there were large effect sizes for both depression and anxiety measures post-treatment in comparison to control, making the IES result at post-test an anomaly.

## Strength of Evidence Using Only the Data Supplied in the RTI-UNC Report

There appears to be differences in how the consistency domain was rated with respect to SOE for PTSD symptom reduction in EMDR compared to other treatments. This section of our review refers to the analysis on the four studies included in the RTI-UNC report. This analysis excludes two important and relevant studies, which are described later in this report. With regards to PTSD symptom reduction, EMDR is rated in the RTI-UNC report as *Inconsistent*. This is based on the heterogeneity of the related studies (*I*^2^= 70%), the direction of the effects and the magnitude of these effects. Examination of the impact of CT on PTSD symptom reduction suggests that there is even higher heterogeneity (*I*^2^ = 79.6%), as shown on Table G-2. However, rather than *Inconsistent*, the evidence was labeled as *Some Inconsistency*. The annotation of this table indicates that the ‘*Direction of effects were consistent; magnitude of effects ranged from very large to small’* (p. G-4). Similar annotations were made in Tables G-1, G-13 resulting in studies with high heterogeneity obtaining ratings of *Consistent* or *Some Inconsistency*.

These annotations have not been applied to the analysis of EMDR. With regards to impact on PTSD symptom reduction, while the heterogeneity of EMDR results is high (*I^2^=* 70%), this is lower than the same measure for CT mentioned above. Further, the direction of the effects from EMDR studies is consistent and the magnitude of these effects ranged from ‘almost small to very large,’ which is similar to related results for CT. This suggests that the consistency domain for EMDR on PTSD symptom reduction should have been moved from *Inconsistent* to *Some Inconsistency*, to ensure uniformity in rating across therapies.

A change of the consistency domain would mean that the domains for PTSD symptom reduction following EMDR would be comparable to that for CT across all measures. Therefore the SOE for EMDR for PTSD symptom reduction should have been moderate rather than low.

It may have been argued that this annotation may not apply to the EMDR results with regards to symptom reduction as one of the studies (Carlson et al., 1998) had a confidence interval where the lower point falls below zero. However, two of the studies in CBT-Mixed Interventions (McDonagh et al., 2005; Johnson et al., 2011) have their confidence intervals falling below zero, and this intervention is still rated as consistent. Further, if the outcome measure analyzed for the Carlson et al. (1998) study was altered as suggested above from the IES to M-PTSD, then none of the EMDR studies would have had the lower point of the confidence interval falls below zero.

## Omissions of Randomized Controlled Trials Relevant to the Research Questions

An additional error in the analysis that occurred in the RTI-UNC report was the failure to include two studies relevant to the issue of whether EMDR leads to more symptom reduction than a control condition. The report purports to assess, as its first research question, the effectiveness of psychological treatments “compared with wait list, usual care (as defined by the study), no intervention, or a placebo,” (pES-5). However, a study by van der Kolk et al. (2007) was omitted. This study assessed three treatment conditions. Participants were randomized to either EMDR or SSRI treatment condition, or a placebo control. This study is cited in the report, however, it is inexplicably missing from the meta-analysis that investigates mean changes in PTSD symptoms for EMDR vs. control comparisons. As placebo is clearly a control condition it should have been included.

This omission cannot be justified on a basis of methodological procedures because other studies that included multiple arms were utilized in more than one place in order to answer key questions. For example, Marks et al. (1998) appears in Table 9 when discussing coping skills trials, and again in Table 13, looking at the efficacy of exposure trials (Jonas et al., 2013). This suggests that there is no methodological issue that would result in the exclusion of the van der Kolk et al. (2007) data. The inclusion of this study into the analysis would change the conclusions on the SOE in the report. When we calculated the new confidence interval it was from -1.56 to -0.37, which is better precision than CPT. Heterogeneity also improved from the analysis of the four studies and continued to be better than CPT or CT.

Another important study omitted from the meta-analysis was published in 2015 (van den Berg et al., 2015). A problem with the APA guidelines is that they were based on the review by RTI-UNC published in 2013, however, the APA guidelines were published in 2017. This means that while readers may believe they are reading 2017 guidelines, they are actually reading guidelines that are 4 years out of date. Three recent randomized control trials (Capezzani et al., 2013; van den Berg et al., 2015; Acarturk et al., 2016) that support EMDR as evidence based are not considered in these conclusions. One study in particular, by van den Berg et al. (2015) meets a high methodological standard. Indeed, in the RTI-UNC appendices this study is highlighted. The APA committee in reviewing the RTI-UNC findings acknowledged that the addition of this study to the analysis was likely to narrow the confidence interval and therefore impact on precision and would also improve consistency. “*If a new meta-analysis were to be done… the confidence interval would be narrower and it is possible that the SOE might be upgraded from low to medium as a result.*” (Appendix p. F-11). However, seemingly paradoxically, after highlighting the impact of the addition of this study, they then conclude that there is insufficient evidence to determine whether the study would change the recommendation for EMDR. In contrast to this view, it is later purported that if the effect size stayed at medium/large, and given the increased sample size of including this study then the overall SOE for EMDR would probably change.

Actually testing this proposition is not difficult nor particularly time consuming. Again, we used Comprehensive Meta-Analysis Software and input the same effect sizes reported from Figure 17 in the RTI-UNC report but added CAPS scores and confidence intervals from the studies of van der Kolk and van den Berg. The results are presented in **Table 1**. The effect size remained large SMD = -0.89 (-1.34, -0.44). The precision improved to a confidence interval difference of just 0.9. Using the RTI-UNC own guidelines of assessing SOE, EMDR is doing better than both CPT and CT in both consistency and precision. In fact, it is closer to mixed CBT in precision than CPT or CT. Even more compelling is the heterogeneity, which at 66% is better than mixed CBT, CT, and CP. The total N is also substantial at 284. Following, it is not possible from a science point of view to rate CPT and CT higher in SOE than EMDR.

**Table 1:** Comparative statistics on effect size, precision, and consistency analysis including changes when all relevant EMDR studies are included with appropriate comprehensive measures.

Treatment	PTSD symptom reduction	Difference	Heterogeneity
Cognitive processing therapy	SMD -1.40 (-1.95, -0.85)	1.10	87%
Cognitive therapy	SMD -1.22 (-1.91, -0.53)	1.38	80%
CBT-mixed	SMD -1.09 (-1.4, -0.78)	0.62	87%
EMDR (original report using IES for Carlson)	SMD -1.08 (-1.83, -0.33)	1.50	70%
EMDR (using M-PTSD for Carlson)	SMD -1.28 (-1.81, 0.74)	1.07	48%
EMDR with van der Kolk and van der Berg and using IES for Carlson	SMD -0.89 (-1.34, -0.44)	0.90	66%
EMDR with van der Kolk and van der Berg and using M-PTSD for Carlson	SMD-0.99 (-1.41, -0.58)	0.93	57%

*SMD, standard mean difference; IES, Impact of Events Scale; M-PTSD, Mississippi Scale for Combat Related PTSD.*

**FIGURE 1:**
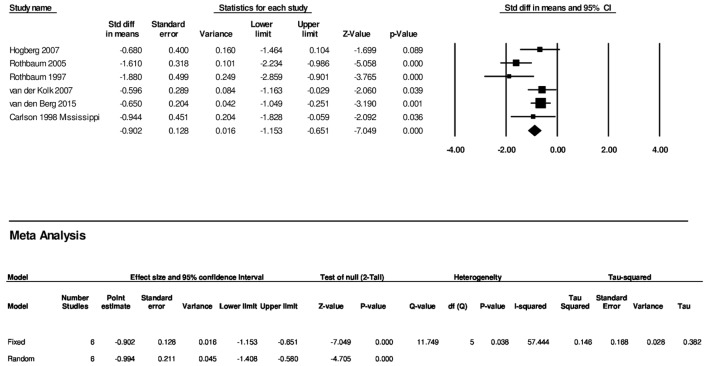
Results of the meta-analysis using all appropriate studies and measures.

Finally redoing the analysis for all six studies that compared EMDR to a control condition and using the more appropriate M-PTSD measure for the Carlson study the SMD is -0.99 and the confidence interval is from -1.41 to -0.58 (*I*^2^= 57%) (see **Figure 1**). This is the best reflection of the state of the literature today. This is the result that should have been used by the APA. This data means that consistency for EMDR is better than CT, CPT and mixed CBT and EMDR has more precision than CT or CPT.

## Papers Inappropriately Included in the Analysis

In examining the papers included from the analysis in the RTI-UNC review, there appear to be errors made in the inclusion of certain studies to the analysis of evidence. An example of this is the inclusion of Taylor et al. (2003), despite several significant validity concerns and concerns regarding the interpretation regarding psychometric properties.

In Table E1 of the RTI-UNC paper, there is a category that examines whether the participant groups in the study were equivalent at baseline. On page E-21, this category for the study by Taylor et al. (2003) was rated as yes. However, no pre-treatment test scores analysis for treatment conditions is reported. The only pre-treatment analysis reported suggests no significant differences between dropouts and completers—regarding demographics and primary measures of interest. Furthermore, Figure 2 indicates that the participants in the exposure group reported less symptoms than those in the EMDR group at pre-treatment (Taylor et al., 2003). The confidence intervals on the bar graph show the mean score for the exposure group was outside the standard error of the EMDR group at pre-treatment for hyperarousal, re-experiencing, and avoidance symptoms.

The bias in the Taylor et al. (2003) study is further inflated as it relied on a treatment completer analysis rather than an intent-to-treat analysis. This is critical as while participants in the EMDR condition had more severe symptoms to begin with, the other CBT condition had a higher dropout rate (11% greater), resulting in an elevated chance of systematic bias.

An additional error in the rater’s assessment of this study was the judgment that the providers of the therapy were masked. However, logic asserts that this assessment is not possible in a design comparing two psychological treatments. Given these errors in the risk of bias the Taylor et al. (2003) study should have been reclassified as high and the study excluded.

The results of the Taylor et al. (2003) study is at odds with other more methodologically sound studies. Removing this study changes the interpretation of the RTI-UNC report with regards to EMDR and PTSD symptom change. The conclusion that all studies ‘…*found a greater reduction in PTSD symptom scores for EMDR than for comparators’* (p. 67) still stands. However, Taylor et al.’s (2003) exclusion alters the effect size for ‘*PTSD symptom reduction for EMDR compared with relaxation’* (p. F-73) and ‘*Loss of PTSD diagnosis at 3-month follow-up for EMDR compared with relaxation’* (p. F-74), in favor of EMDR. The exclusion of this study also impacts the data comparing relaxation to exposure therapy.

## Papers Inappropriately Excluded From the Analysis

In examining the papers excluded from the analysis in the RTI-UNC report (Jonas et al., 2013), there appears to be errors made in the exclusion of some studies from the analysis. Research by Lee et al. (2002) was assessed as a high risk of bias. However, as explained below, there appear to be errors in the examination of the results of this study.

In Table E1 on the RTI-UNC paper, there is a category that examines whether the participant groups in the study were equivalent at baseline. On page E-13, this category for the study by Lee et al. (2002) was rated as unclear. However, page 1077 of the Lee et al. (2002) article reports,

“Independent t-tests were used to investigate differences between the groups on pre-treatment measures. No differences were found for the IES [t(22) = 0.11, p = 91], BDI [t(22) = 1.05, p = 0.31], SI-PTSD [t(22) = 1.63, p = 0.12], or MMPI-K [t(22) = 1.31, p = 0.21]. Therefore, the groups appeared to be equivalent on major variables.”

Therefore, the raters made an error in asserting that the paper was not clear on whether there were differences at baseline. This is in sharp contrast to the Taylor et al. (2003) study where no baseline comparison data was analyzed.

The raters of Lee et al.’s (2002) study also marked it down saying that that the differential attrition data was unclear. However, the study clearly indicates that 24 participants entered the study, 12 were assigned EMDR and 12 were assigned to CBT, with three people dropping out, leaving 21 completers. On page 1075, it is stated that 21 participants completed the study, 11 for stress inoculation with prolonged exposure and 10 from EMDR. The article then describes how one of the EMDR non-completer was sent to prison. It does not make sense that the raters can claim that the attrition is not clear.

Given the above two errors, the risk of bias in the study deserves to be reclassified from high risk of bias to moderate. This inclusion strengthens the evidence base for a reduction in PTSD symptoms and for the loss of diagnosis for EMDR.

If correctly applying the RTI-UNC criteria to assess the evidence for EMDR to treat PTSD the APA should consider seven randomized controlled trials. Of these trials, four investigated EMDR compared to another manualized treatment and a waitlist or other minimal intervention control (Carlson et al., 1998; Rothbaum et al., 2005; van der Kolk et al., 2007; van den Berg et al., 2015), two compared EMDR treatment to a waitlist control only (Rothbaum, 1997; Högberg et al., 2008), and one trial compared EMDR to another manualized treatment only (Lee et al., 2002).

## Lack Of Attention to Follow Up Data

In the RTI-UNC analysis, it states “*Our meta-analysis (Figure 17) found greater reduction in PTSD symptoms for EMDR than for controls…. Treatment gains were maintained for studies reporting follow up at 3, 6, or 9 months* (p. 67).” This statement ignores the considerable data that EMDR treatment gains are maintained far beyond end of treatment time points. At the very least the follow up study on the Högberg et al. (2008) data, which reported treatment gains for EMDR were maintained at 35 months, should have been mentioned. Other data, such as that presented in Wilson et al. (1995, 1997) papers, should also have been included. In this study, the researchers show that treatment gains made following just three EMDR sessions were maintained at follow-up (15 months) with large effect sizes.

## Exclusion of Studies Treating PTSD Where Severity of Symptoms Did Not Meet the Full Diagnostic Criteria

The outcomes from the RTI-UNC review are based on studies with individuals who meet the Diagnostic and Statistical Manual of Mental Disorders (DSM) criteria for PTSD (typically DSM-IV). However, there is a longstanding debate in the literature with regards to the classification of mental health disorders, including PTSD (Haslam, 2003). Classification systems, such as the DSM, support a categorical classification system where by specific number of symptoms are provided in order to meet a diagnosis. Alternatively, a dimensional approach involves viewing mental health problems on a continuum without the arguable arbitrary cut of point that exists in a categorical classification (Brown and Barlow, 2005). The acknowledgment of the dimensional approach, and the inclusion of related studies, would significantly broaden the scope for the analysis and lead to more accurate data that is more meaningful to the practitioner (Luyten and Blatt, 2007). Typically practitioners would not refuse treatment to someone who wanted help in dealing with their trauma because they failed to meet all the diagnostic criteria from the DSM. Such a position is untenable especially as the diagnostic criteria changes over time and with different diagnostic systems. In the end, it is a science question. That is, where is the evidence of a differential effect of treatment on participants who make criteria and those who don’t? With respect to PTSD at least one study reported no differences in the effect size on the outcome measures for those who met diagnostic criteria and those who did not (Wilson et al., 1995). Therefore to dismiss such studies as “wrong population” as cited in the RTI-UNC report lacks practical as well perhaps scientific credibility. There are three randomized controlled trials that were dismissed because of this position by the committee (Vaughan et al., 1994; Wilson et al., 1995, 1997; Scheck et al., 1998). All had solid methodology including assessing PTSD symptoms with a structured interview. These trials all found strong effects for EMDR over comparative treatments. There exclusion weakens the generalizability of the guidelines.

## Response From the APA with Regards to this Review

Prior to publication of the APA Practice Guidelines Development Panel for the Treatment of PTSD, an earlier version of this paper was submitted to the committee. The response of the Development Panel was to either ignore the main points of this paper or to respond with inaccurate information (Selected Representative Comments on PTSD Draft Document 1-24-17, American Psychological Association, forwarded as a personal communication by H. Kurtzman, 7 April 2017). For example, in response to the inappropriate measure issue in the Carlson et al. (1998) study, they stated that the IES was used as it is ‘a more standard instrument’ (p. 67) and that the M-PTSD was not used in any other study. However, as noted in this review they used the M-PTSD over the IES in other parts of their review. Regarding the failure to include the van der Kolk et al. (2007) study and the clear inappropriate inclusion of Taylor et al. (2003) study the panel simply failed to give any comments or responded by suggesting that no error had been made in with regards to the use of these studies. They do not directly address to the issues that were raised.

## Conclusion

The APA guidelines are utilized worldwide and the accuracy of the document and the data it contains is crucial. This review highlights some serious inaccuracies regarding the way studies were handled in the statistical review of papers particularly with respect to evidence concerning EMDR. Therefore, the subsequent conclusions of the draft guidelines are flawed. Such failure to acknowledge errors explains why the proposed 2017 guidelines are at odds with other best practice guidelines from other countries and international based guidelines such as the World health Organization in 2013 (World Health Organization, 2013).

## Author Contributions

SD conducted all statistical analysis and reviewed the final version of the manuscript. CL initiated the writing of the article, provided the initial review of the RTI-UNC article and communicated directly with American Psychological Association regarding the content of this paper and relevant documents. Both authors contributed to the reviewing of the relevant papers and the studies they contained, and reviewing the draft versions of this manuscript.

## Conflict of Interest Statement

CL has received fees for providing training in trauma therapies and personality disorders. The other author declares that the research was conducted in the absence of any commercial or financial relationships that could be construed as a potential conflict of interest.
